# Case Report: Anal canal duplication associated with anorectal stenosis—A rare presentation

**DOI:** 10.3389/fped.2022.955845

**Published:** 2022-11-29

**Authors:** Zhen Zheng, Dong Xiao, Junxiang Wang, Haozhong Xu, Jianxiong Mao, Xiuliang Wang

**Affiliations:** ^1^ Department of General Surgery, Shenzhen Children's Hospital, Shenzhen, China; ^2^ College of Medicine, Shantou University, Shantou, China

**Keywords:** case reports, congenital malformations, alimentary tract duplication, constipation, anal canal duplication, megacolon

## Abstract

Anal canal duplication is a rare gastrointestinal malformation characterized by extra anal orifices at 6 o'clock in the lithotomy position. To date, there have been only 110 reported cases. The purpose of this study is to contribute two infant cases, one of which is associated with anorectal stenosis, which has never been described.

## Introduction

Anal canal duplication (ACD) is the rarest malformation of the distal alimentary tract, usually defined as “cases with an additional blind anal orifice, sagittally positioned, exhibiting the same histological features as normal anal canal” (1). The pathology criteria were set by Ochiai et al. (2) in 2002, as follows: the presence of (1) squamous epithelial cells in the caudal end, (2) transitional epithelium in the cranial end, and (3) smooth muscle cells in the lesion wall. There were only about 110 cases described in the literature, with a female-to-male ratio of 15:1 (3–8). Patients usually present with a second opening in the midline, at 6 o’clock behind the true anus (9, 10). Rare cases such as 5 o’clock position orifice or anal canal triplication were also reported (7, 11). Infant patients were often asymptomatic. Some patients, mostly adults and children older than 5 years, complained of constipation, mucous drainage, or perineal abscess. Some patients were misdiagnosed with complex anal fistula and suffered from abscesses being incised and drained many times (6, 12–15). Anorectal malformation, anal duplication cyst, malrotation, sacrococcygeal teratoma, myelomeningocele, tethered cord, congenital heart defects, cleft lip/palate, and genitourinary anomalies were found in approximately 25% of the cases, with presacral mass accounting for 50% of the total (3, 9). Complete excision and simple mucosectomy have been demonstrated with satisfactory outcomes (16).

We describe two additional infant cases: case 1 presented with refractory constipation, and case 2 was asymptomatic. A barium enema of case 1 revealed anorectal stenosis and mega-rectosigmoid. Two patients underwent surgery, and the pathology confirmed the diagnosis.

### Case 1

**Figure 1:**
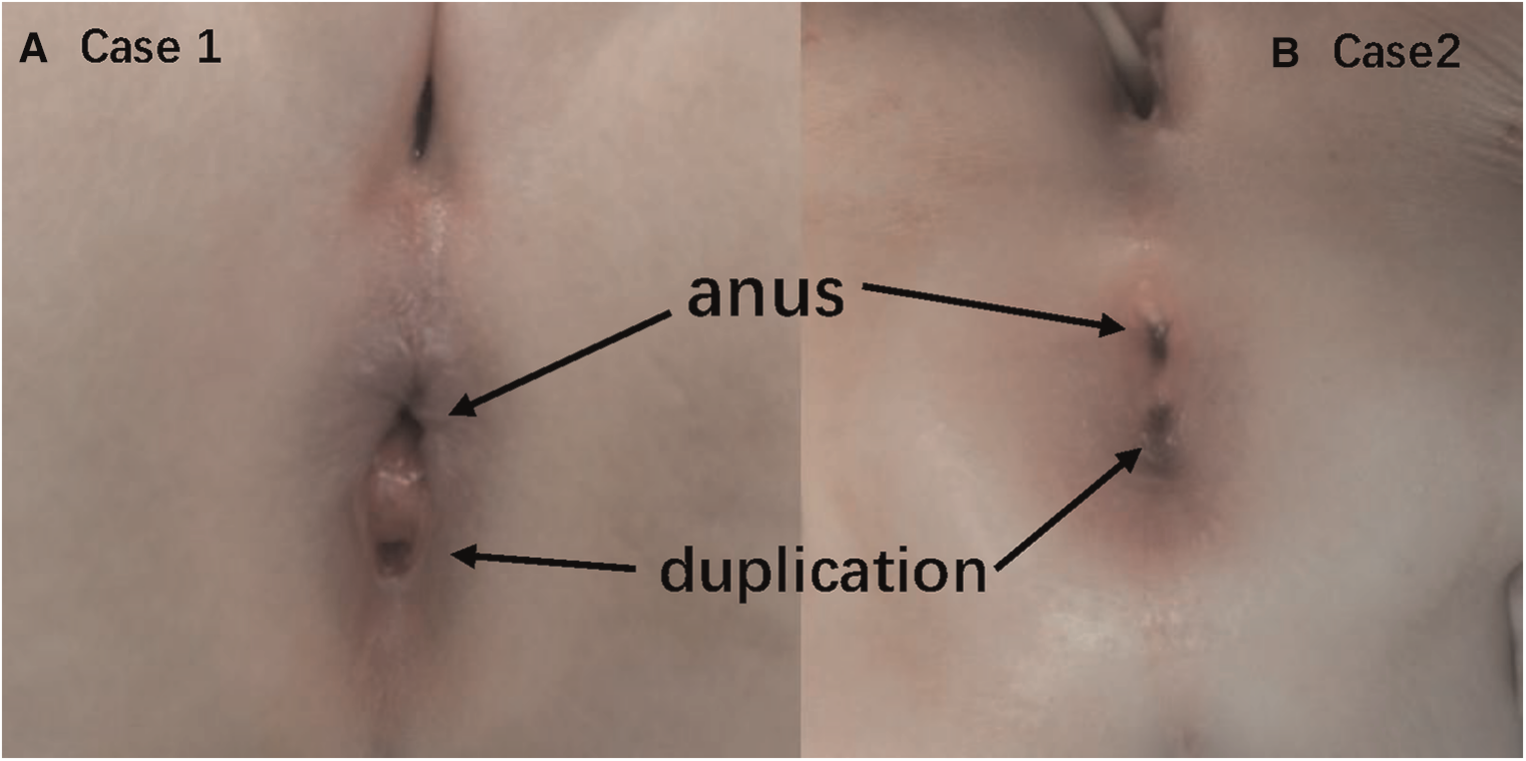
Duplication situating in the midline, at 6 o’clock direction in the lithotomy position.

**Figure 2:**
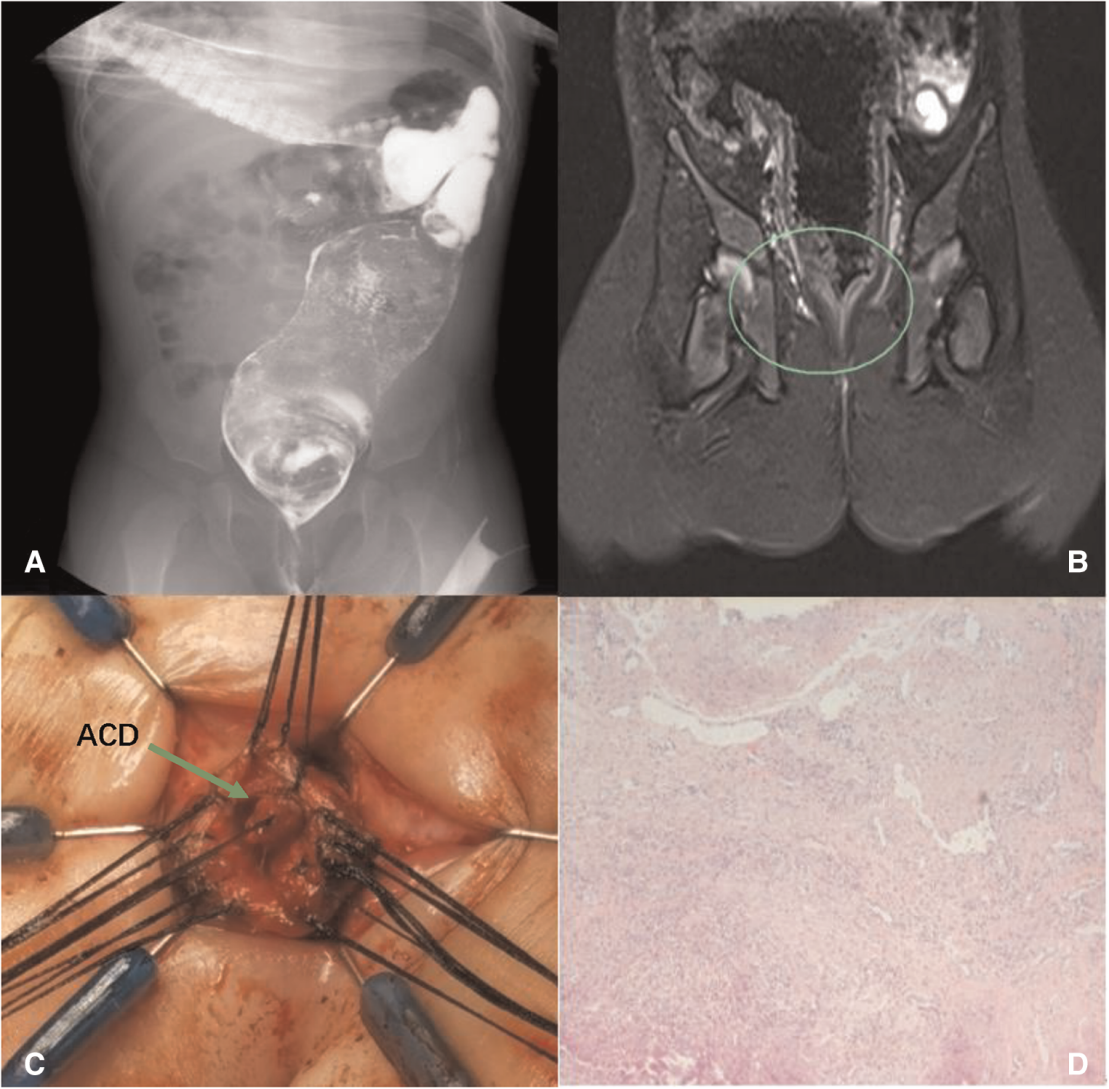
Case 1: dilated rectum and sigmoid colon (**A**); rectal stenosis presented in MRI (**B**); complete removal of the ACD (**C**); histology showing squamous epithelium, fibers, blood vessels, and smooth muscle tissue (**D**).

An 8-month-old baby was referred for refractory constipation. She was born full-term and fully breast-fed since birth, without other medical issues. As she began complementary food, she developed progressive constipation. Treatment such as laxatives and enema was given to aid in defecation, but the condition aggravated. A posterior anal orifice was identified on physical examination (Figure 1A). Barium fistulography and contrast enema revealed a narrowed distal rectum at the level of the fifth sacral vertebra. The proximal rectum and sigmoid colon were significantly dilated, with the widest part being about 44 mm (Figure 2A). No dilation was observed in the descending colon and transverse colon. Magnetic resonance imaging (MRI) demonstrated a bird's beak-like appearance of the distal rectum and no presacral mass or spinal abnormality. The duplication appeared as a line of high-signal lesions communicating with the skin (Figure 2B).

Removal of the entire ACD and anoplasty were performed in a prone position. After anesthesia, we used a urethral catheter to confirm the depths of ACD and the normal anus. We could put a maximum 10Fr catheter (about 3.3 mm in diameter) through the normal anus, while only a 6Fr (about 2 mm in diameter) catheter could be introduced into the ACD tube with a depth of 3 mm. The existence of ACD led to rectum stenosis. The operation could be divided into two parts: (1) ACD dissection and (2) treatment of rectal stenosis and anoplasty.

We started with a dissection of ACD ventral mucosa and found a 20-mm-long blind tubular structure that started in the rectum and ended near the dentate line, sharing a mucosal wall with the normal anus and rectum but without communication (Figuer 2C). The blind tube was meticulously dissected circumferentially, and the mucosal wall of the rectum side was left completely intact. The rectal stenosis extended 20 mm, and we performed a transanal pull-through procedure for the rectum with full thickness in the range of the sphincter. Then, we resected the stenosed part of the rectum and performed an anastomosis between the rectum and dentate line. In consideration of the possibility of spontaneous recovery and to reduce surgical trauma, the dilated proximal rectum and sigmoid colon were left untreated.

**Figure 3:**
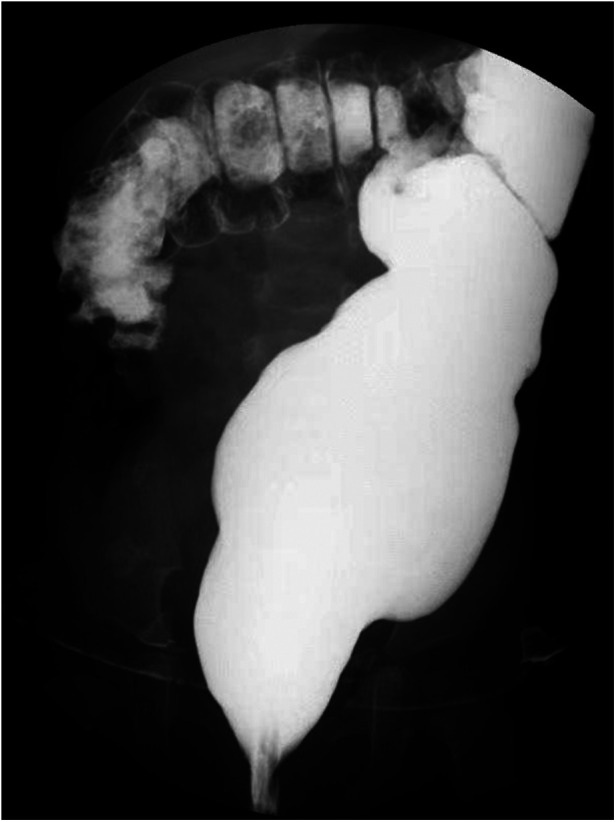
Case 1: barium edema 4 months after surgery.

Histology confirmed the diagnosis of ACD and showed the specimen covered with squamous epithelium, fibers, blood vessels, and smooth muscle tissue (Figure 2D). Hegar’s dilatation was given after surgery. Re-examination of the barium enema 4 months after surgery showed that the distal rectum was 6 mm and the mega-rectosigmoid progressed, with the widest part being about 60 mm (Figure 3). However, in physical examination, 17 mm Hegar’s dilator can go smoothly through the anus. The patient can discharge yellow and soft stools once a day without assistance.

### Case 2

**Figure 4:**
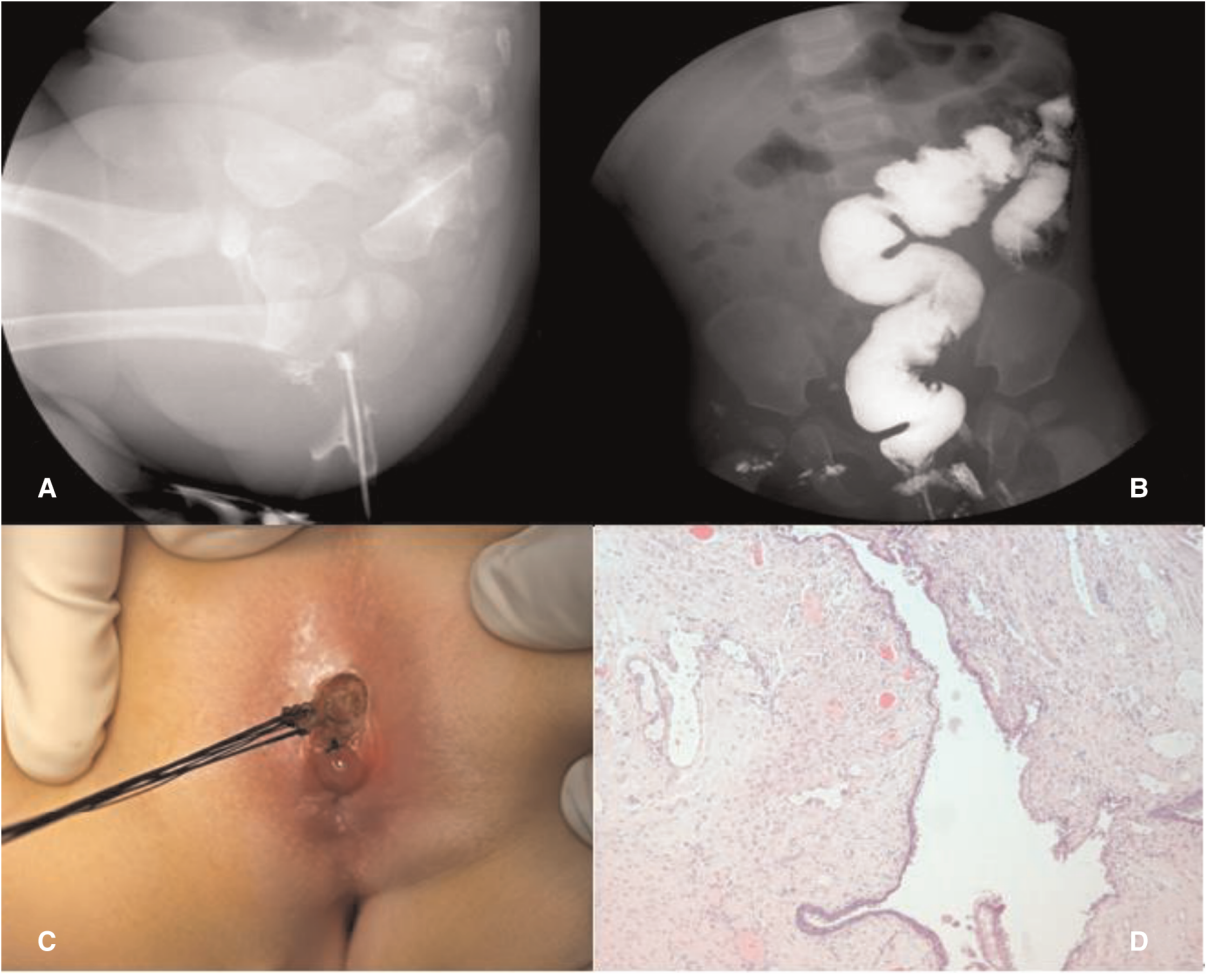
Case 2: Barium fistulography of ACD (**A**); barium enema (**B**); simple mucosectomy (**C**); histology showing squamous epithelium (**D**).

An 11-month-old girl was found to have a posterior anal orifice since birth (Figure 1B). She was asymptomatic and had full-term birth, without other medical problems. Barium fistulography and contrast enema showed a 5-mm-long and 4-mm-diameter tubular area with no communication with the rectum (Figure 4A). No obvious abnormality was observed in the size and shape of the colon and rectum (Figure 4B). MRI revealed no spinal abnormalities or presacral mass.

A simple mucosectomy was performed in the prone position. The duplication was completely resected and sent for pathological examination, with the posterior rectal wall left completely intact (Figure 4C). Histology confirmed the diagnosis of ACD and showed that the specimen was covered with squamous epithelium (Figure 4D); no complication was reported during the 11-month follow-up.

## Discussion

Anal canal duplication is a rare malformation with poorly understood etiology and less defined clinical management criteria. Patients are always asymptomatic or just with local symptoms around the perianal region. Differential diagnosis should be carefully evaluated between ACD with perianal fistula and perianal abscess. Comorbidities were found in 25% of the patients, of which presacral masses were the most common, accounting for 50%, and anorectal malformations (ARMs) account for 5% (3, 5). We report a case associated with anorectal stenosis, accompanying secondary mega-rectosigmoid, which has never been described before.

Early diagnosis is crucial but easy to miss in neonates (17). Our patient was suspected of having ACD and anorectal stenosis 8 months after birth; mega-rectosigmoid most likely occurred due to delayed diagnosis and treatment. The diagnosis should be made when a second perineal opening appears behind the normal anus at 6 o’clock. The diagnosis of concomitant malformations is also important because of their direct impact on the surgical strategy. Auxiliary examinations such as fistulography and MRI are useful tools for ACD diagnosis. Contrast enema can describe the diameter and length of the duplication and, more importantly, the communication with the rectum. MRI can inform the relationship between ACD and adjacent soft tissue like the anal sphincter and striated muscle. A key role of MRI is to find other abnormalities such as presacral mass, intrasacral meningocele, or duplicated rectum. Methylene blue detection can be performed to check whether there is communication between ACD and the anus.

The etiology and the reason for the female incidence dominance remain poorly understood due to the complexity of embryonic development. Interestingly, male patients make up a larger proportion of patients with an imperforate anus (18). Previous studies believe that the formation of ACD is closely related to the process of urorectal septum development; however, a further investigation should be performed (19). Direct observation of embryonic development is admissible, but the difficulty in creating ACD animal models is a problem.

The prognosis for surgery is favorable, but the necessity is controversial (16). Akova et al. proposed that asymptomatic patients who do not undergo surgery might remain free of symptoms (5). This could be due to the small number of follow-up cases (only 2) and the short average follow-up period (3.5 ± 1.0 years). The statistical data of 110 cases reported in the literature show that patients who develop symptoms are mostly adults or children older than 5 years (3, 6, 9, 10). Moreover, it has been proposed that the possibility of developing symptoms and complications increases with age, patients will develop abdominal pain, perianal pain, constipation, and other atypical symptoms, and the situation will deteriorate as they become older (6, 12, 14). This may relate to an increase in stool volume as the patient ages. It is also unknown whether the condition has an impact on mental development. Furthermore, research indicated that ACD patients might be predisposed to colloid carcinoma (20). All cases we described underwent surgery, and the follow-ups were all uneventful. Given the excellent outcomes of surgery, we believe that surgery is the best option to treat ACD before it progresses into bothersome symptoms and reduces the risk of developing colloid cancer.

The clinical features of ACD, the anatomic relationship with the anal sphincter, and its associated malformations are important determinants of the surgical approach (21). For cases associated with ARMs and presacral mass, the posterior sagittal approach is recommended (22). In our case, the ACD was located in the rectum and no presacral malformations were associated. For complete resection of the stenotic rectum and to reduce peripheral neuromuscular injury, we performed a transanal pull-through procedure for the rectum with full thickness in the range of the sphincter, which showed good outcomes.

## Conclusion

ACD is a rare malformation with a lack of well-defined clinical care guidelines and a poorly understood etiology. Early clinical suspicion of ACD and association elimination are crucial. Contrast enema and MRI are useful auxiliary examinations for ruling out presacral masses and ARMs. For ACD associated with anorectal stenosis, a transanal pull-through procedure of the rectum with full thickness is recommended with good results.

## Data Availability

The original contributions presented in the study are included in the article/Supplementary Material, further inquiries can be directed to the corresponding author/s.
